# Development of novel waxy bone haemostatic agents composed of biodegradable polymers with osteogenic-enhancing peptides in rabbit models

**DOI:** 10.1093/icvts/ivad170

**Published:** 2023-10-31

**Authors:** Tsukasa Ohno, Hiroto Suenaga, Aika Yamawaki-Ogata, Kei Kanie, Ryuji Kato, Koichiro Uto, Mitsuhiro Ebara, Hideki Ito, Yuji Narita, Akihiko Usui, Masato Mutsuga

**Affiliations:** Department of Cardiac Surgery, Nagoya University Graduate School of Medicine, Tokai National Higher Education and Research System, Nagoya, Japan; Department of Cardiac Surgery, Nagoya University Graduate School of Medicine, Tokai National Higher Education and Research System, Nagoya, Japan; Department of Cardiac Surgery, Nagoya University Graduate School of Medicine, Tokai National Higher Education and Research System, Nagoya, Japan; Department of Basic Medicinal Sciences, Graduate School of Pharmaceutical Sciences, Nagoya University, Tokai National Higher Education and Research System, Nagoya, Japan; Department of Biotechnology and Chemistry, Kindai University, Higashi-Hiroshima, Japan; Department of Basic Medicinal Sciences, Graduate School of Pharmaceutical Sciences, Nagoya University, Tokai National Higher Education and Research System, Nagoya, Japan; Division of Micro-Nano Mechatronics, Institute of Nano-Life-Systems, Institutes of Innovation for Future Society, Nagoya University, Tokai National Higher Education and Research System, Nagoya, Japan; Biomaterials Field, Research Center for Functional Materials, National Institute for Materials Science (NIMS), Tsukuba, Japan; Biomaterials Field, Research Center for Functional Materials, National Institute for Materials Science (NIMS), Tsukuba, Japan; Department of Cardiac Surgery, Nagoya University Graduate School of Medicine, Tokai National Higher Education and Research System, Nagoya, Japan; Department of Cardiac Surgery, Nagoya University Graduate School of Medicine, Tokai National Higher Education and Research System, Nagoya, Japan; Department of Cardiac Surgery, Nagoya University Graduate School of Medicine, Tokai National Higher Education and Research System, Nagoya, Japan; Department of Cardiac Surgery, Nagoya University Graduate School of Medicine, Tokai National Higher Education and Research System, Nagoya, Japan

**Keywords:** Haemostatic agents, Bone wax, Sternum, Biodegradable polymer, Peptides, Bleeding

## Abstract

**OBJECTIVES:**

The use of bone wax (BW) is controversial for sternal haemostasis because it increases the risk of wound infection and inhibits bone healing. We developed new waxy bone haemostatic agents made from biodegradable polymers containing peptides and evaluated them using rabbit models.

**METHODS:**

We designed 2 types of waxy bone haemostatic agents: peptide wax (PW) and non-peptide wax (NPW), which used poly(ε-caprolactone)-based biodegradable polymers with or without an osteogenesis-enhancing peptide, respectively. Rabbits were randomly divided into 4 groups based on treatment with BW, NPW, PW or no treatment. In a tibial defect model, the bleeding amount was measured and bone healing was evaluated by micro-computed tomography over 16 weeks. Bone healing in a median sternotomy model was assessed for 2 weeks using X-ray, micro-computed tomography, histological examination and flexural strength testing.

**RESULTS:**

The textures of PW and NPW (*n* = 12 each) were similar to that of BW and achieved a comparable degree of haemostasis. The crevice area of the sternal fracture line in the BW group was significantly larger than that in other groups (*n* = 10 each). The PW group demonstrated the strongest sternal flexural strength (*n* = 10), with complete tibial healing at 16 weeks. No groups exhibited wound infection, including osteomyelitis.

**CONCLUSIONS:**

Waxy biodegradable haemostatic agents showed satisfactory results in haemostasis and bone healing in rabbit models and may be an effective alternative to BW.

**Figure:**
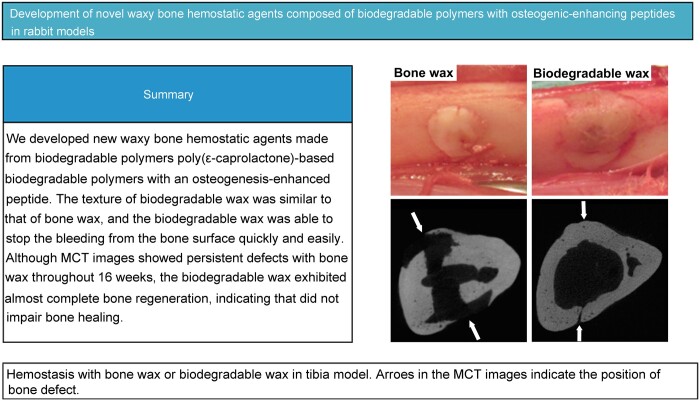

## INTRODUCTION

Full median sternotomy, developed by Ormand Julian in 1957, is the conventional procedure used to enter the mediastinum in cardiovascular surgery [1]. It is estimated that 10 000 cardiothoracic surgeons in 6000 centres globally perform >2 million open-heart operations per year [2]. Inadequate sternal haemostasis leads to poor surgical visibility, and haematoma formation also increases the incidence of bacterial infections and the risk of postoperative mortality and morbidity [3]. Therefore, careful haemostasis with electric coagulation or haemostatic agents is essential for successful surgery [4].

Bone wax (BW) is widely applied for bone haemostasis because it is effective, has a high performance-to-cost ratio and is simple to use. Although BW is an effective haemostatic agent for bone bleeding, it is a non-biodegradable material that is not metabolized or resorbed and remains indefinitely at the site of application [5, 6]. In addition, it has been reported that BW increases infection rates, interferes with bone healing and results in chronic inflammatory reactions and sternal nonunion [6, 7]. Hence, BW is not recommended for sternal application and received a class III recommendation in a guideline for the prevention of mediastinitis [8]. An ideal bone haemostatic agent should have the following properties: a waxy texture, so it can be easily packed into bone marrow; biodegradability and biocompatibility, so it does not impair bone healing; and adequate haemostatic efficacy. In addition, bone haemostatic agents should ideally promote bone regeneration.

Poly(ε-caprolactone) (PCL) and poly(d,l-lactide) (PDLLA) are well-known biodegradable polymers that are widely used in biomedical devices such as bone scaffolds and pins for sternal fixation. Hydroxyapatite (Hap) is also used in medical applications because its mineral composition resembles that of natural bone and it exhibits good osteoconductivity [9]. Recently, short functional peptides have attracted attention for biomedical applications [10, 11]. We previously showed that osteogenic-enhancing peptides promoted the differentiation of mesenchymal stem cells (MSCs) to osteoblasts [12]. In the present study, we describe novel waxy bone haemostatic agents developed using PCL, PDLLA, Hap and osteogenic-enhancing peptides and evaluated their haemostatic and bone healing capability.

## MATERIALS AND METHODS

### Animals and ethics

Seven- to 12-week-old male Japanese white rabbits (1.26–1.56 kg, Slc: JW/CSK, closed colony) were purchased from Japan SLC, Inc. (Hamamatsu, Shizuoka, Japan) and maintained on a regular chow diet under standard conditions. All animal experiments were performed in accordance with the Guide for the Care and Use of Laboratory Animals published by the United States National Institutes of Health (NIH publication No. 85-23, revised 2011) and were approved by the Animal Care and Use Committee of Nagoya University (protocol No. 31168) on 8 March 2019.

### Poly(ε-caprolactone)-based biodegradable polymer

To develop degradable base materials with a soft texture resembling that of BW, we selected a random copolymer composed of d,l-lactide (DLLA), ε-caprolactone (CL) and Hap. Four-armed poly(d,l-lactide-*co*-ε-caprolactone) (PDLLA-PCL) was synthesized by ring-opening polymerization of DLLA and CL from the terminal hydroxyl groups of pentaerythritol using tin octanoate as a catalyst, as described in previous studies [13, 14]. The DLLA/CL ratio was 40/60 (mol%). The structure and molecular weight were estimated by proton nuclear magnetic resonance spectroscopy (JEOL, Tokyo, Japan) and gel permeation chromatography (GPC; JASCO International, Tokyo, Japan). The thermal properties of the obtained polymer were characterized by differential scanning calorimetry (DSC6100, Seiko Instruments, Chiba, Japan). The measurements were conducted from 0 to 120°C at a heating rate of 5°C min^−1^. The viscoelastic properties of the copolymers were evaluated using a rheometer (MCR 301, Anton Paar, Tokyo, Japan) with parallel plate geometry (rotating top plate of 10-mm diameter). Furthermore, equal weights of Hap (50 wt%) and PDLLA-PCL (50 wt%) (in a molten state) were vigorously mixed to create a homogeneous PDLLA-PCL/Hap composite. The texture of this PCL-based biodegradable polymer above the melting temperature (*T*_m_) was achieved by the use of Hap [13].

### Osteogenic-enhancing peptides

Osteogenesis-enhancing peptides were determined by *in silico* and peptide array screening in a previous study [12]. Briefly, several homologous sequences of bone morphogenetic proteins (BMPs), including BMP-2, BMP-4, BMP-6 and BMP-7, were identified by *in silico* screening. Twenty-five candidate peptides were selected from the homologous regions of 9 consecutive amino acid sequences. Then, a direct cell assay using a peptide array was employed to select the osteogenic-enhancing peptide TLVNSVNSK, which enhanced osteogenic cell-selective proliferation and osteogenic differentiation of MSCs. The TLVNSVNSK peptide was prepared using a conventional solid-phase chemical synthesis method (GL Biochem (Shanghai) Ltd., Shanghai, China) and had a purity of over 90%.

### Preparation of waxy bone haemostatic agents

We prepared 2 types of waxy bone haemostatic agents: peptide wax (PW) and non-peptide wax (NPW), which were PCL-based biodegradable polymers with or without an osteogenic-enhancing peptide, respectively. PW was obtained by kneading the osteogenic-enhancing peptide (TLVNSVNSK) at 0.2% (w/w) into the PCL-based biodegradable polymer. Supplementary Material, Fig. S1A shows the appearance of each haemostatic agent, specifically BW, NPW and PW.

All rabbits were anaesthetized using intramuscular ketamine (10 mg/kg) and xylazine (1–3 mg/kg) and maintained on isoflurane. All animals received mefenamic acid (25 mg) after surgery for analgesia. The animals were returned to the recovery room after surgery for postoperative care.

### Tibia model

To evaluate the amount of bone marrow bleeding after the use of haemostatic agents, a cortical bone defect model was established by creating bone defects on each tibia. Twenty-four rabbits were randomly divided into 4 groups. Twelve tibiae of 6 rabbits per group were pierced with a 2-mm drill and treated with or without haemostatic agents. Bleeding from the bone defects was controlled using NPW, PW, BW or a beeswax-based haemostat (Ethicon, Inc, Somerville, NJ). The sham group was treated without any haemostatic agents. Equal amounts (0.02 g) of each haemostatic agent were applied to cover the tibial holes. Sterilized dry gauze was then applied to the bone defect for 3 min (Supplementary Material, Fig. S1B), and the amount of bleeding was measured using a digital scale with a minimum display of 0.01 g (HT-120, A&D Company, Tokyo, Japan). Measurements were rounded off to the second decimal place and expressed to 2 significant digits. Finally, the surgical wounds were closed using 3–0 VICRYL^®^ (Ethicon, Inc., Somerville, NJ). Sixteen weeks postoperatively, the animals were sacrificed by an overdose of intravenous potassium chloride under general anaesthesia using intramuscular ketamine and xylazine, and the tibias were harvested.

### Sternal model

Sternotomy was performed with a circular saw under anaesthesia with 1–2% isoflurane. Forty rabbits were randomly divided into 4 groups based on haemostatic treatment with NPW, PW, BW or no haemostatic agent as a control (*n* = 10, respectively). Equal amounts (0.2 g) of the haemostatic agents were applied to cover each cut bone surface. The sternum was fixed by 2–0 braid silk suture (Supplementary Material, Fig. S1C), and subcutaneous tissue was closed with 4–0 VICRYL (Ethicon, Inc., Somerville, NJ). Two weeks postoperatively, the animals were sacrificed by an overdose of intravenous potassium chloride under general anaesthesia using intramuscular ketamine and xylazine, and the sterna were harvested. The sterna of 10 rabbits without sternotomy were harvested as a control group. The enucleated sterna were then osteotomized at the third and fourth joints; the former pieces were subjected to mechanical fracture strength testing, and the latter were fixed immediately in 10% phosphate-buffered formaldehyde for histological analysis.

### X-ray and micro-computed tomography analysis

X-ray and micro-computed tomography (MCT) evaluations were performed on all sterna and tibiae. The crevice area on sternal X-rays was quantified by ImageJ software (National Institutes of Health, Bethesda, MD, USA). MCT analysis was performed on a SkyScan 1176 Technical Speciﬁcations 64 MCT scanner (BRUKER, Kontich, Belgium). MCT images of tibiae were obtained at the middle of the defective area, and those of the sternum were obtained at the middle of the third joint. Sternal quality was quantitatively assessed on MCT scans by analysing Hounsfield units (HU) on 10 randomly selected horizontal slices using CT-analyser software version 1.13 (Bruker).

### Three-point flexural strength tests

Fresh sternal specimens in each group were used for three-point flexural strength testing. The test was performed with an AUTOGRAPH AGS-J 500N (SHIMADZU Corporation, Kyoto, Japan) using a three-point evaluation with a 0.2-mm/s crosshead speed (Supplementary Material, Fig. S1D). The data were analysed by TRAPEZIUM2 data processing software (SHIMADZU Corporation).

### Histology

Specimens were obtained from the fourth joint of the sternum, washed with saline and fixed with 4% paraformaldehyde (PFA, Fujifilm Wako Pire Chemical Corporation, Osaka, Japan) at 4°C for 2 days. They were then decalcified with ethylenediamine tetraacetic acid (Sigma-Aldrich, St. Louis, MO, USA) for about 2 months before embedding in paraffin. Specimens were cut into 5-µm-thick horizontal sections and stained with haematoxylin–eosin. All microscopic assessments were performed using an FSX-100 microscope (OLYMPUS, Tokyo, Japan).

### Statistical analysis

Statistical analyses of the bleeding amount, crevice area, HU value and flexural strength were performed using Tukey’s multiple comparisons test. *P* < 0.05 was considered to be statistically significant. All statistical analyses were performed using SPSS for Windows, version 24 (SPSS, Chicago, IL, USA). Data are expressed as median and interquartile range (IQR).

## RESULTS

### Characterization of poly(d,l-lactide-*co*-ε-caprolactone)

PDLLA-PCL was obtained as a viscous solid, and its number average molecular weight was estimated to be 37100 by GPC calibrated with a polyethylene glycol standard. GPC also showed that the copolymer possessed a relatively narrow molecular weight distribution (polydispersity index: weight-average molecular weight/number average molecular weight = 1.44). The DLLA/CL ratio in the copolymer was determined by proton nuclear magnetic resonance to be 63.9/36.1 mol% (Supplementary Material, Fig. S2A). Differential scanning calorimetry showed that the copolymer had a *T*_m_ of around 37°C, which was lower than that of BW (Supplementary Material, Fig. S2B and C). To provide insight into the texture of the copolymer, we carried out a rheological analysis. For PDLLA-PCL, the loss modulus (G″) was higher than the storage modulus (*G*′) at 37°C. The values of *G*′ and *G*″ for PDLLA-PCL were much higher than those for BW (Supplementary Material, Fig. S2D and E), suggesting that the copolymer behaves as a viscous liquid above *T*_m_ and is stickier than BW. Hap was then mixed with molten PDLLA-PCL to create a waxy biodegradable polymer. The stickiness of PDLLA-PCL was significantly reduced by mixing with Hap, which made the resulting substance easier to handle, even above *T*_m_.

### Evaluation of the bleeding amount

**Figure 1:**
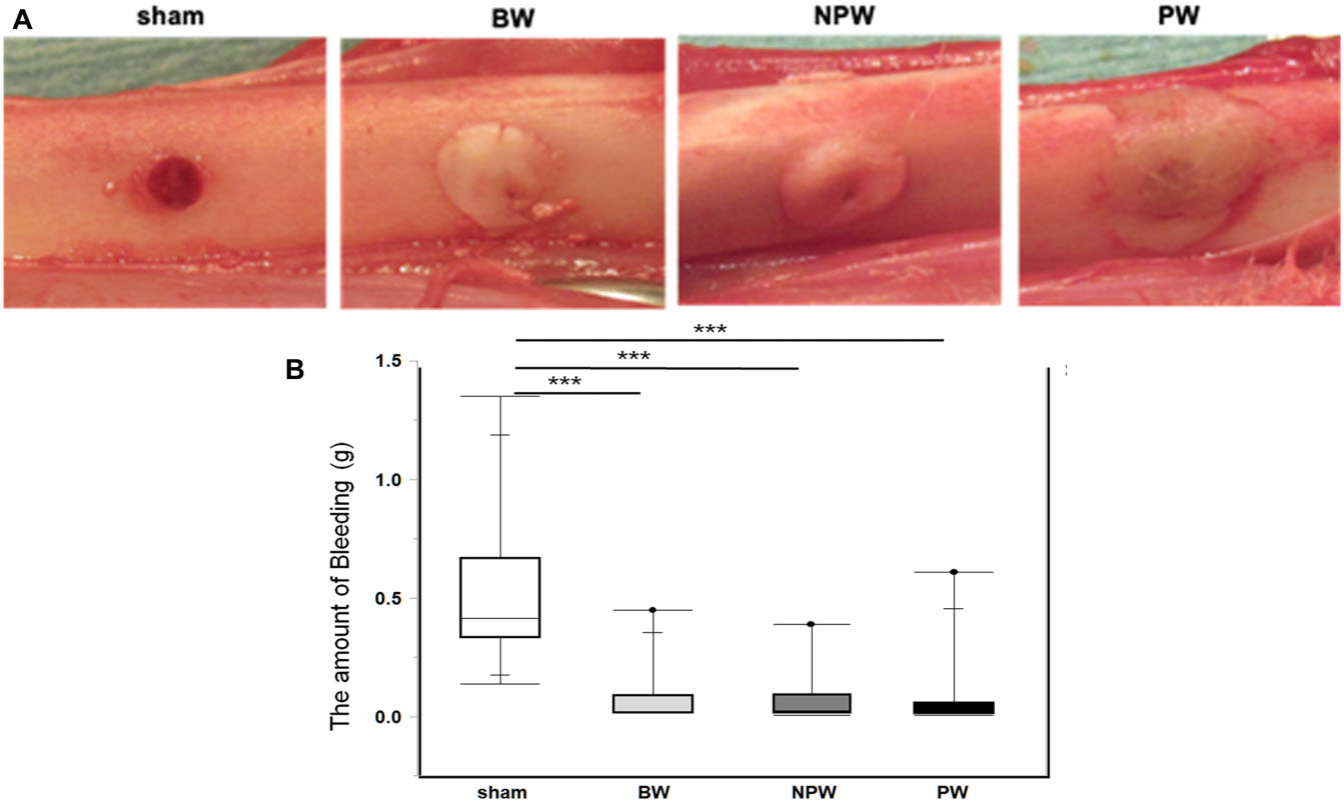
Haemostasis with or without wax in a tibial model. (**A**) Representative images at the time of haemostasis in each group. (**B**) The amount of bleeding was larger in the sham group than in the other groups (*n* = 12 each). ***P < 0.001 versus sham group assessed by Tukey’s multiple comparisons test.

The waxy haemostatic agents PW and NPW were malleable and could be applied to bone surfaces with the fingertips as easily as BW. Bleeding from the bone surfaces was quickly and easily controlled in the NPW and PW groups, and the haemostatic action of these substances showed similar efficacy to that of BW (Fig. 1A). The median amount of bleeding in each group was as follows: NPW group, 0.03 (IQR 0.02–0.09) g; PW group, 0.04 (IQR 0.01–0.05) g; BW group, 0.02 (IQR 0.02–0.07) g; sham group, 0.4 (IQR 0.36–0.60) g. The sham group exhibited the greatest amount of bleeding (*P* < 0.001), with no significant difference between the PW, NPW and BW groups (Fig. 1B).

### Sternal assessment

No postoperative sternal infection or mediastinitis was observed in any of the rabbits during the study. Radiograms of the sternum are shown in Supplementary Material, Fig. S3. Sterna treated with BW showed large crevices at the implantation sites. In contrast, those treated with PW and NPW showed adequate coaptation that was similar to that in the sham group (Supplementary Material, Fig. S3A). The crevice area in the BW group was significantly larger [median 26.5 (IQR 25.5–28.0)%] than that in any other group [NPW group, median 11 (IQR 9.1–13.4)%; PW group, median 9.6 (IQR 7.9–10.3)%; sham group, median 10.2 (IQR 9.3–11.0)%; *P* < 0.001, Supplementary Material, Fig. S3B].

**Figure 2:**
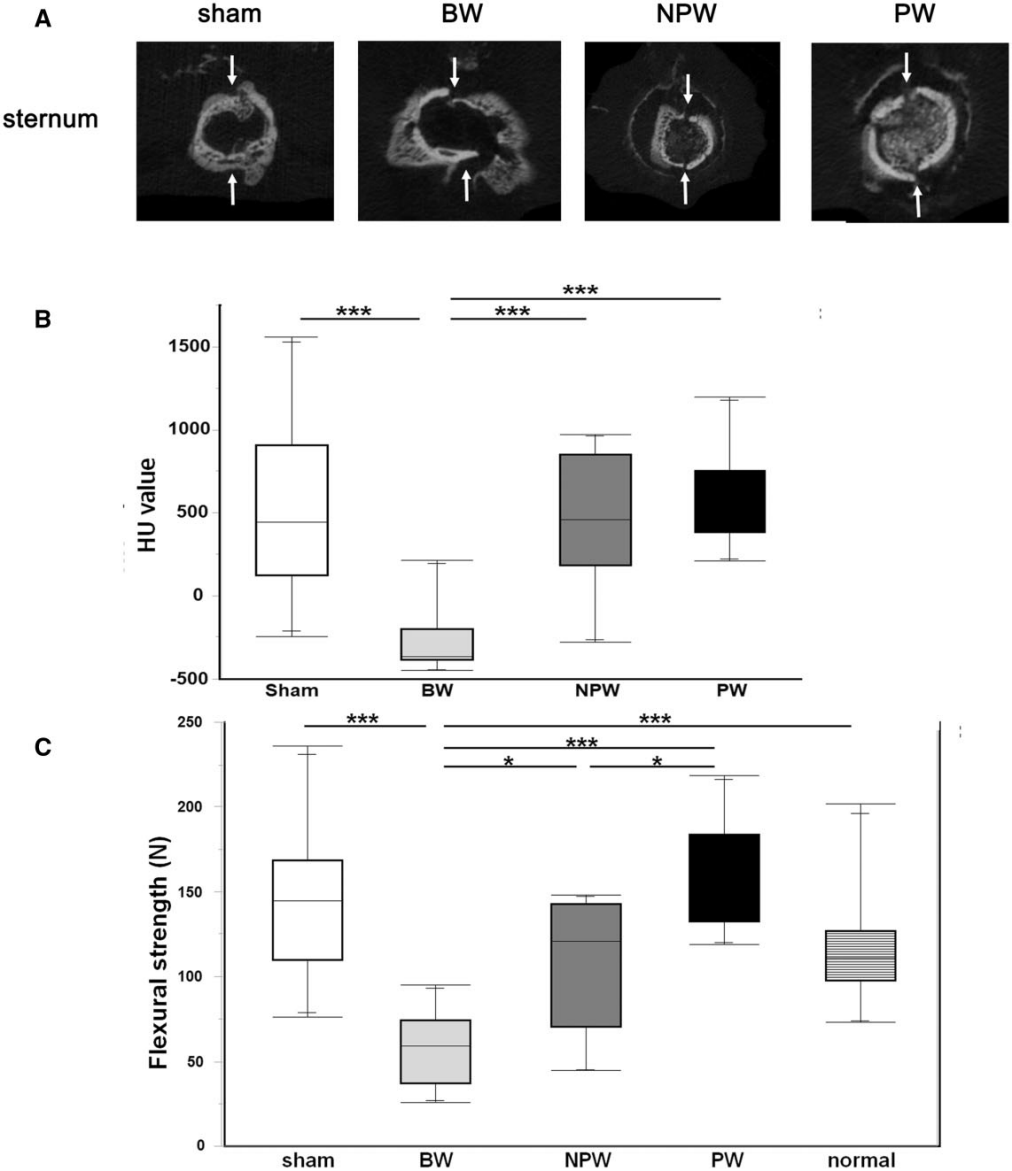
Sternum at 2 weeks after surgery. (**A**) Representative micro-computed tomography images of each group. White arrows indicate the incision line. (**B**) Hounsfield unit values measured by micro-computed tomography (*n* = 10 each). (**C**) Mechanical strength measured by three-point flexural strength testing. The sternum was weakest in the bone wax group and was stronger in the peptide wax group than in the non-peptide wax group (*n* = 10 each). *P < 0.05, ***P < 0.001 assessed by Tukey’s multiple comparisons test.

MCT images are shown in Fig. 2A. Sternal fractures were observed in the BW group. The median HU value was −368.5 (IQR −386.8 to −281.8) HU in the BW group, which was significantly lower than that in any other group (Fig. 2B, *P* < 0.001). There were no significant differences between the other groups [NPW group versus PW group versus sham group, median 459 (IQR 290.0–827.3) HU versus median 560 (IQR 407.5–673.8) HU versus median 440.5 (IQR 165.8–732.5) HU, respectively]. In the three-point flexural strength test, the median result in the BW group was 59.1 (IQR 41.1–70.9) N, which indicates significant weakness compared to the other groups [NPW group, median 120.9 (IQR 84.7–137.7) N; PW group, median 152.1 (IQR 134.9–177.1) N; sham, median 144.7 (IQR 117.9–159.1) N; BW versus NPW, *P* < 0.05; BW versus PW, *P* < 0.001; BW versus sham, *P* < 0.001; BW versus normal, *P* < 0.05; Fig. 2C]. The strength in the NPW group, median 111.0 (IQR 105.8–121.0) N, was similar to those in the sham and normal groups. Moreover, the PW group exhibited significantly greater strength than the NPW group (*P* < 0.05).

### Histological findings

**Figure 3:**
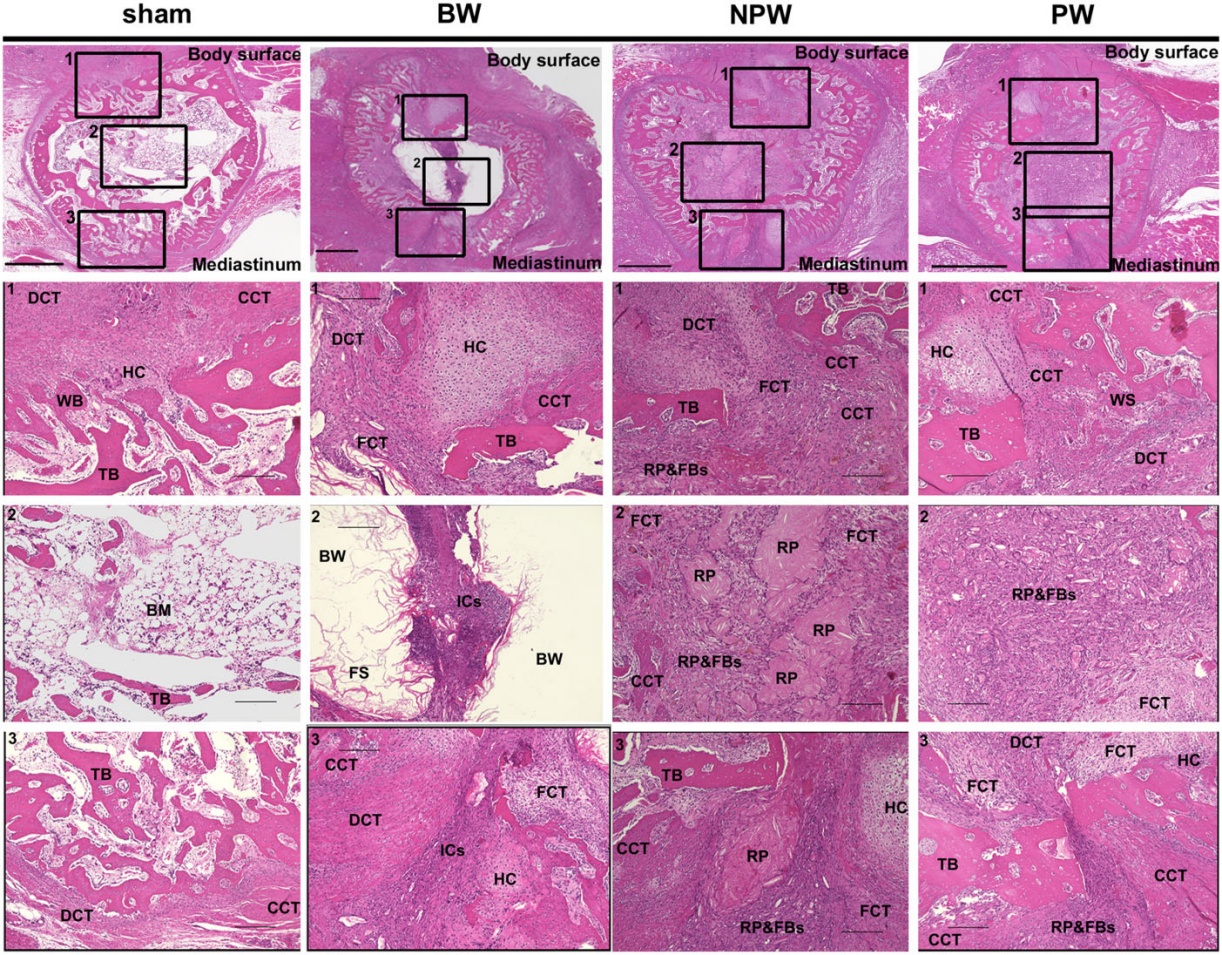
Haematoxylin–eosin staining of sternal bone in each group at 2 weeks postoperatively. Scale bar = 100 μm. BM: bone marrow; CCT: calcified cartilaginous tissue; DCT: dense connective tissue; FBs: fibroblast-like cells; FCT: fibrous connective tissue; FS: fibrotic scarring; HC: hyaline cartilage; ICs: inflammatory cells; RP: residual polymer; TB: trabecular bone; WB: woven bone.

Results of histological analysis of the sternum are shown in Fig. 3. The injured area in the sham group had a bone structure similar to the normal sternum, with the presence of bone marrow and trabecular bone. On the other hand, the medullary cavity in the BW group exhibited residual BW, fibrosis and many inflammatory cells, including lymphocytes and macrophages. In addition, the cortical bone was surrounded by hyaline cartilage, fibrotic connective tissue and inflammatory cells. Fragmented residual polymers were observed in the NPW group, while these were almost absorbed in the PW group. These residual polymers were surrounded by inflammatory cells and fibroblast-like cells. Fibrous connective tissue, dense connective tissue, hyaline cartilage and calcified cartilaginous tissue were also present at the cortical bone surgical site in both the NPW and NP groups.

### Long-term evaluation of tibiae

**Figure 4:**
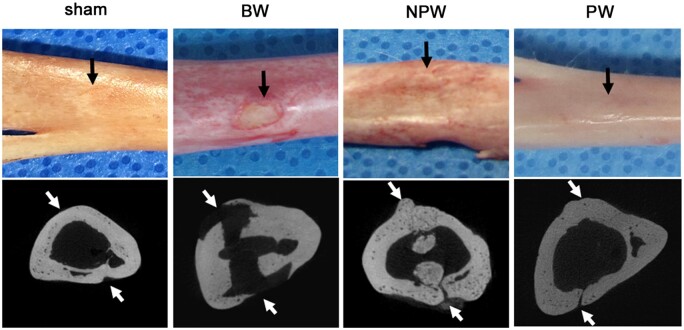
Representative images of tibiae at 16 weeks after surgery. The upper panel shows morphological images, and the lower panel shows micro-computed tomography images. Black and white arrows show the positions of bone defects.

None of the groups in the tibia model exhibited wound infection. The NPW, PW and sham groups showed almost complete bone regeneration at 16 weeks after surgery (Fig. 4), while MCT images showed persistent defects in the BW group.

## DISCUSSION

Post-sternotomy haemostatic agents should be flexible enough to evenly cover the rough surface of the bone and also be viscoelastic enough to remain embedded. BW, which is conventionally applied, has these properties. Using the biodegradable polymers PDLLA, PCL and Hap, we developed new waxy haemostatic agents that mimicked the texture of BW. These agents softened around 37°C and, like BW, could be kneaded with the fingers. Moreover, they could be easily applied to the bone surface and to the bone marrow cavity, which has fine gaps, and showed stable haemostatic ability due to their viscoelastic property. There was no significant difference in haemostatic ability between BW and the waxy haemostatic agents, indicating that the latter are an effective alternative to BW for bone marrow bleeding.

Haemostatic agents used for bone marrow bleeding should also avoid inhibiting bone healing. In this study, MCT scans of the sternum showed that the area of low HU value surrounding the divided cortical bone was larger in the BW group than in the other groups. This suggests that the non-absorbed BW remnant impaired sternal ossification. Additionally, sterna treated with BW showed significantly lower bone strength than the other 3 groups. Sixteen-week tibial observation showed that while BW inhibited bone regeneration, PW and NPW resulted in almost complete bone regeneration, indicating that the haemostatic agents did not impair bone healing. An important difference between BW and haemostatic agents is that the latter are biodegradable.

Several biodegradable haemostatic materials for bone marrow haemostasis have been marketed in the USA. Ostene (Baxter International Inc., Chicago, IL, USA), which is a waxy haemostatic agent composed of a high-molecular-weight poly(oxyethylene)/poly(oxypropylene)/poly(oxyethylene) triblock copolymer, was shown to be absorbed between 24 and 48 h after surgery, and unlike BW, it did not inhibit bone healing in animal models [5, 15]. In addition, histomorphometric analysis showed that BoneSeal (Hemostasis, LLC, White Bear Lake, MN, USA), which consists of PLA and Hap, resulted in new bone formation in animal experiments [16]. These haemostatic agents consisting of biodegradable polymers are indeed advantageous for bone healing compared to BW. However, these studies provided insufficient data on the flexural strength of healed sterna, including comparison to intact sterna. Additionally, it was not mentioned whether the textures of these materials were similar to that of BW, or if these agents accelerated bone regeneration. In this study, we demonstrated that sterna treated with NPW was significantly stiffer than those treated with BW, and PW resulted in even greater stiffness than NPW, indicating the efficacy of the additional peptides included in PW. A previous study showing that these peptides enhanced osteogenesis indicated that they may accelerate bone healing and regeneration; as amino acid sequences of BMPs, they not only enhance the osteogenic differentiation of MSCs but also inhibit fibroblast proliferation [12]. This evidence demonstrates that the osteogenesis-enhancing peptides do not induce abnormal cell proliferation or fibrous scarring but instead lead to natural bone regeneration, resulting in greater bone strength.

### Limitations

This study has several limitations. First, although the haemostatic agents were completely absorbed at 16 weeks, we did not attempt to optimize the absorption period to promote faster bone healing. Additionally, the amount of these haemostatic agents needed to obtain sufficient haemostasis is still unknown. Second, it has been reported that the incidences of postoperative sternal wound infection and mediastinitis range from 1.4% to 3.3% [17–19], and the use of BW is associated with an increased risk of infection [20]. We observed no cases of infection with either BW or the new haemostatic agents. Theoretically, a biodegradable material should decrease the risk of surgical site infection, because no foreign material remains in the implanted site. A study using an animal femur model showed that a bioabsorbable phospholipid gel was associated with a significantly lower infection rate than polymethyl methacrylate, non-bioabsorbable material [21]. Third, we did not evaluate the process of long-term bone healing in the sternum model. However, the flexural strength test 2 weeks postoperatively showed that sterna treated with the haemostatic agents had the same strength as sham and normal sterna, indicating that sternal healing might be achieved in 2 weeks. Detailed analysis, including that of haemostatic agent degradation over time, should be performed to better characterize biomaterials. Fourth, although this study suggests that the osteogenic-enhancing peptides accelerated bone healing, the *in vivo* pharmacokinetics and pharmacodynamics of these peptides are unclear. Therefore, the ideal composition of the osteo-enhancing peptides remains to be elucidated. Further investigations are necessary to resolve these questions prior to clinical application.

## CONCLUSIONS

We newly developed waxy haemostatic agents using the biodegradable polymers PDLLA, PCL and Hap. These agents were biocompatible and were as effective as BW in achieving bone marrow haemostasis. Furthermore, the haemostatic agent with osteogenic-enhancing peptides increased the strength of the sternum at 2 weeks after surgery.

## Supplementary Material

ivad170_Supplementary_Data

## Data Availability

Data collected for the study will be made available by the corresponding author upon reasonable request after publication.

## ABBREVIATIONS

BMPs: Bone morphogenetic proteins
BW: Bone wax
CL: ε-Caprolactone
DLLA: d,l-Lactide
*G*′: Storage modulus
*G*″: Loss modulus
GPC: Gel permeation chromatography
Hap: Hydroxyapatite
HU: Hounsfield units
NPW: Non-peptide wax
MSCs: Mesenchymal stem cells
PCL: Poly(ε-caprolactone)
PDLLA: Poly(d,l-lactide)
PDLLA-PCL: Poly(d,l-lactide-co-ε-caprolactone)
PW: Peptide wax
*T*_m_: Melting temperature
