# Supplementary material for: Development of novel waxy bone haemostatic agents composed of biodegradable polymers with osteogenic-enhancing peptides in rabbit models
Source: Interdiscip Cardiovasc Thorac Surg. 2023 Oct 31;37(5):ivad170. doi: 10.1093/icvts/ivad170 (PMC10639036; doi:10.1093/icvts/ivad170)
Supplement: ivad170_Supplementary_Data [file ivad170_supplementary_data.pdf]

Figure S1: Photographs of PCL-based biodegradable polymers, and illustrations of *in vivo* model and three-point flexural strength testing.

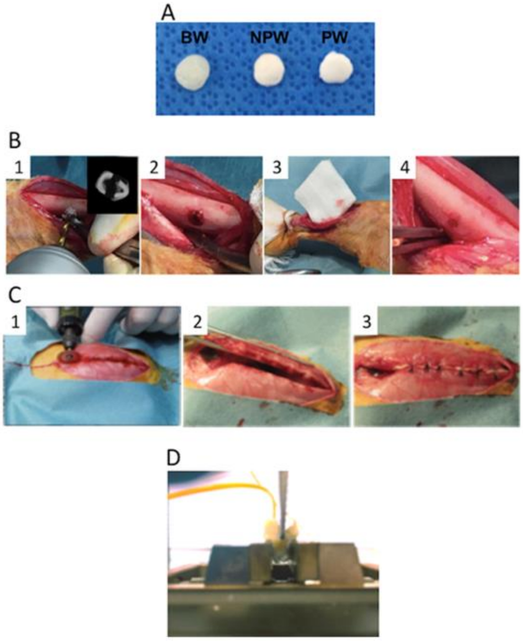

(A) The appearances of the hemostatic materials analyzed in this study. (B) In the tibia model, a hole was made in each rabbit's tibia using a drill (B1), and bleeding was stopped with or without hemostatic agents (B2). Measurement of the amount of bleeding from the defect area (B3). Image after hemostasis with the hemostatic agents shows in (B4). (C) In the sternum model, full sternotomy was performed with a circular saw (C1). Hemostatic agents were applied to the surface after cutting (C2). The sternum was closed by 2-0 braid silk suture (C3). (D) Flexural strength testing of sternal specimens was performed.

Figure S2: Characterization of PDLLA-PCL.

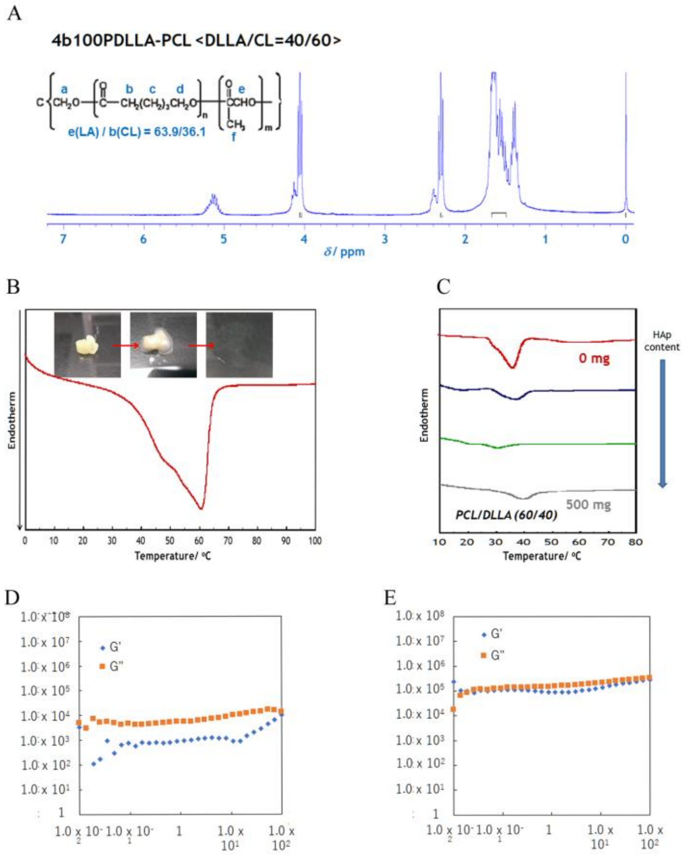

(A) ^{1}H NMR spectra of DLLA and CL in the copolymer. The DLLA/CL ratio was 63.9/36.1 mol%. The DSC curve shows that the Tm of bone wax is around 37℃ (B) and that of PDLLA-PCL was maintained around the same temperature by mixing PCL with DLLA at a 60/40 ratio (C). The rheological properties of BW (D) and PDLLA-PCL (E). Both G' and G" of BW are between $1.0*10^2$ and $1.0*10^4$ Pa at 37℃, and those of PDLLA-PCL were maintained between 10^4 and 10^5 Pa at 37℃. G': storage modulus, G": loss modulus.

Figure S3: Radiographic analysis of the sternum.

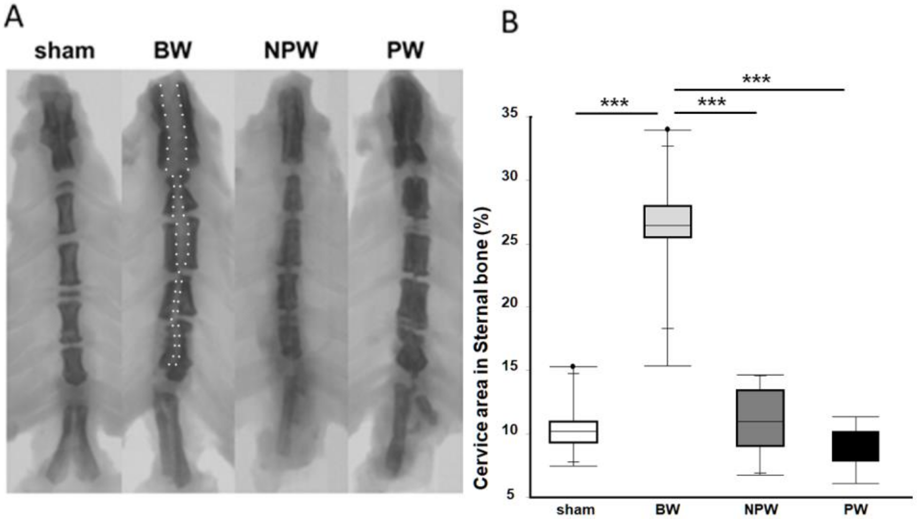

(A) Representative X-rays were obtained 2 weeks after sternotomy. (B) Crevice areas in the manubrium and body of the sternum were analyzed by Image J software. The crevice area was larger in the BW group than in the other groups. *** P < 0.001 vs BW group assessed by Kruskal Wallis by Tukey’s honestly significant difference test. The error bars signify the standard error of the mean.
